# Gamabufotalin Inhibits Osteoclastgenesis and Counteracts Estrogen-Deficient Bone Loss in Mice by Suppressing RANKL-Induced NF-κB and ERK/MAPK Pathways

**DOI:** 10.3389/fphar.2021.629968

**Published:** 2021-04-23

**Authors:** Kaiqiang Sun, Jian Zhu, Yi Deng, Ximing Xu, Fanqi Kong, Xiaofei Sun, Le Huan, Changzhen Ren, Jingchuan Sun, Jiangang Shi

**Affiliations:** ^1^ Department of Spine Surgery, Changzheng Hospital, Naval Medical University, Shanghai, China; ^2^ Department of Pharmacy, Changzheng Hospital, Naval Medical University, Shanghai, China; ^3^ Department of Cardiology, Changzheng Hospital, Naval Medical University, Shanghai, China

**Keywords:** gamabufotalin, OVX model, osteoporosis, osteoclast, ERK/MAPK, receptor activator of nuclear factor kappa-B ligand, TRAF6

## Abstract

Osteolytic bone disease is a condition of imbalanced bone homeostasis, characterized mainly by excessive bone-resorptive activity, which could predispose these populations, such as the old and postmenopausal women, to developing high risk of skeletal fragility and fracture. The nature of bone homeostasis is the coordination between the osteoblasts (OBs) and osteoclasts (OCs). Abnormal activation of osteoclasts (OCs) could compromise the bone homeostasis, constantly followed by a clutch of osteolytic diseases, including postmenopausal osteoporosis, osteoarthritis, and rheumatoid arthritis. Thus, it is imperatively urgent to explore effective medical interventions for patients. The traditional Chinese medicine (TCM) gamabufotalin (CS-6) is a newly identified natural product from Chansu and has been utilized for oncologic therapies owing to its good clinical efficacy with less adverse events. Previous study suggested that CS-6 could be a novel anti-osteoporotic agent. Nevertheless, whether CS-6 suppresses RANK-(receptor activator of nuclear factor-κ B ligand)/TRAF6 (TNF receptor-associated factor 6)-mediated downstream signaling activation in OCs, as well as the effects of CS-6 on OC differentiation *in vivo*, remains elusive. Therefore, in this present study, we aimed to explore the biological effects of CS-6 on osteoclastogenesis and RANKL-induced activation of related signaling pathways, and further to examine the potential therapeutic application in estrogen-deficient bone loss in the mice model. The results of *in vitro* experiment showed that CS-6 can inhibit RANKL-induced OC formation and the ability of bone resorption in a dose-dependent manner at both the early and late stages of osteoclastogenesis. The gene expression of OC-related key genes such as tartrate-resistant acid phosphatase (TRAP), CTSK, DC-STAMP, MMP9, and β3 integrin was evidently reduced. In addition, CS-6 could mitigate the systemic estrogen-dependent bone loss and pro-inframammary cytokines in mice *in vivo*. The molecular mechanism analysis suggested that CS-6 can suppress RANKL/TRAF6-induced early activation of NF-κB and ERK/MAPK signaling pathways, which consequently suppressed the transcription activity of c-Fos and NFATc1. Taken together, this present study provided ample evidence that CS-6 has the promise to become a therapeutic candidate in treating osteolytic conditions mediated by elevated OC formation and bone resorption.

## Introduction

Bone tissue is highly dynamic, where bone remodeling occurs consistently *via* the formation of new bone and the elimination of old bone, and this homeostasis relies mainly on reciprocally delicate balance between osteoclast (OBs) and osteoclast (OCs) (Kim et al., 2018; He et al., 2017). In general, the bone remodeling is composed of three consecutive phases: OCs initiating bone resorption; catabolism conditioning the transition to anabolism condition; and OBs triggering bone formation. Each of these phases is under the control of the nervous system and endocrine system of the body (Negishi-Koga and Takayanagi, 2012). However, abnormal activation of OCs may result in an imbalance of OCs and OBs, which may compromise the homeostasis and lead to osteolytic bone diseases such as osteoporosis and inflammatory bone destruction (Boyle et al., 2003; Cho et al., 2020). Thus, prophylactic agents with the ability to limit the excessive bone resorption resulting from overactivated OCs are still imperatively required for the population with osteolytic diseases (Chen et al., 2019).

The initiation of OC differentiation is the pre-requirement for bone-resorptive response. In general, OCs are originated from bone marrow–derived monocyte–macrophage (BMM) lineage, and the macrophage colony-stimulating factor (MCSF) and receptor activator of nuclear factor-κ B ligand (RANKL) are the two indispensable regulators during the formation of functional OCs (Lagasse and Weissman, 1997; Kong et al., 1999; Wang et al., 2020). The MCSF ensures normal proliferation and survival of BMM cells *via* activating its receptor c-Fms and downstream Akt and ERK1/2 signaling pathways, while RANKL is indispensable for promoting the differentiation of OCs by its receptor RANK which could interact with TNF receptor-associated factor 6 (TRAF6) and activate a series of downstream cascades, including mitogen-activated protein kinases (MAPK) and NF-κB pathways (Biskobing et al., 1995; Zhou et al., 2016; Kim et al., 2017). Subsequently, the activated MAPK and NF-κB pathways can activate osteoclastogenic transcription factors, mainly including T-cell cytoplasmic 1 (NFATc1) and c-Fos, and then simulate the expression of tartrate-resistant acid phosphatase (TRAP), cathepsin K (CTSK), matrix metalloproteinase (MMP9), dendritic cell–specific transmembrane protein (DC-STAMP), and other genes requisite for osteoclastogenesis (Chiu and Ritchlin, 2016; Zhou et al., 2016; Kim et al., 2017). As a result, agents that could target those key transcription factors or genes may ameliorate the excessive activated OCs and facilitate the treatment of bone loss–related diseases.

Natural products are an important treasure trove, most of which can provide promising agents with superior activity and less toxicity in clinical research and practice (He et al., 2019). The traditional Chinese medicine (TCM) Chansu has been reported to possess various pharmacological properties, such as anti-inflammatory effect, and has widely been used in clinical cancer (Meng et al., 2009; Zhakeer et al., 2017). Chansu is a dried product of the skin and parotid venom glands from the Asiatic toad (*Bufo gargarizans*) (Yu et al., 2016). Previous study reported that as a member of Chansu, bufalin showed promisingly therapeutic effects in treating inflammatory diseases and refractory pain from bone cancer (Ji et al., 2017). Gamabufotalin (CS-6) is a newly identified natural product from Chansu and has been used for anticancer treatment owing to its good biological stability *in vivo* (Yu et al., 2014; Tang et al., 2016; Zhang et al., 2016). However, in addition to its growth inhibitory effects in multiple myeloma *in vitro*, CS-6 also had additive therapeutic effects on myeloma-induced lytic bone destruction, as reported by Yu et al. (Yu et al., 2014). This could be ascribed to the suppressive effect of CS-6 on OCs differentiation and the secretion of pro-inflammatory factors. Nevertheless, whether CS-6 could block the activation of RANK/TRAF6 cascades in OCs, as well as the effects of CS-6 on OCs differentiation, remains elusive.

Therefore, the purpose of this present study was to investigate the effect of CS-6 on osteoclastogenesis and RANKL-induced signaling pathway, and the results showed that CS-6 can inhibit OC formation and bone resorptive activity in a dose-dependent manner at both the early and late stages *in vitro*. In addition, CS-6 could mitigate the estrogen-dependent bone loss and the secretion of pro-inframammary cytokines in mice *in vivo*. The molecular analysis suggested that CS-6 can suppress downstream activation of NF-κB and ERK/MAPK signaling pathways triggered by RANKL/TRAF6, which consequently attenuated the induction of c-Fos and NFATc1. Taken together, this present study provided the evidence that it is possible that CS-6 could become a potential new therapeutic candidate in treating osteolytic diseases mediated by overactivated OCs.

## Methods and Materials

### Animals Ethics Statement

The protocol of animal experiment was in accordance with the rules of the Ethics Committee of the Naval Medical University Institutional Animal Care and the Guide for the Care and Use of Laboratory Animals of the National Institute Health (United States). All the mice used in this study were C57BL/6 mice. The mice were bred in temperature-controlled, specific pathogen-free conditions with a 12-h light/dark cycle, and had free access to water and standard mouse chow.

### Media and Reagents

The cell culture medium (alpha modification of Eagle’s medium, α-MEM), penicillin/streptomycin (P/S), and fetal bovine serum (FBS) were all purchased from Gibco-Technology (Thermo Fisher Institute of Biotechnology, MD, United States). The cell viability testing kit (CCK-8) was obtained from Dojindo Institute of Biotechnology (Kumamoto, Japan). Recombinant mouse M-CSF and RANKL were obtained from R&D Biotechnology Company (Minneapolis, MN, United States), and gamabufotalin (CS-6) was obtained from MedChemExpress (HY-N0883, MCE, Shanghai, China, purity >99.96%), and was dissolved in dimethyl sulfoxide (DMSO; Beyotime Institute of Biotechnology, Jiangsu, China) with stock mother solution concentration of 10 mM in −80°C. The TRAP staining kit was from Sigma-Aldrich Institute of Biotechnology (St. Louis, MO, United States). Specific primary antibodies mainly included NF-kB p65 (D14E12, #8242, Cell Signaling Technology, Inc., three Trask Lane Danvers, United States), p-p65 (Ser536; #3033, Cell Signaling Technology, Inc., three Trask Lane Danvers, United States), Ik-Ba (380682, 35 kDa; Zenbio, Chengdu, China, 1:1,000), p-Ik-Ba (340776,35 kDa; Zenbio, Chengdu, China, 1:1,000), ERK1/2 (201245-4A4, 42/44 kDa; Zenbio, Chengdu, China, 1:1,000), p-ERK1/2 (301245, 42/44 kDa; Zenbio, Chengdu, China, 1:1,000), p38 (200782, 43 kDa; Zenbio, Chengdu, China, 1:500), p-p38 (310069, 43 kDa; Zenbio, Chengdu, China, 1:1,000), JNK (381100, 46/54 kDa; Zenbio, Chengdu, China, 1:1,000), p-JNK (380556, 46/54 kDa; Zenbio, Chengdu, China, 1:1,000), c-Fos (9F6, #2250, Cell Signaling Technology, Inc., three Trask Lane Danvers, United States), NFATc1 (#8032; D15F1, Cell Signaling Technology, Inc., three Trask Lane Danvers, United States), MMP9 (ab228402, 1:1,000, Abcam, Cambridge, MA), TRAF6 (385803, 58 kDa; Zenbio, Chengdu, China, 1:1,000), GAPDH (#5174, D16H11, Cell Signaling Technology, Inc., three Trask Lane Danvers, United States), and Histon H3 (250182, 15 kDa; Zenbio, Chengdu, China, 1:1,000). Fluorescently labeled secondary goat anti-rabbit IgG antibody (IRDye 800CW; ab216773), Phalloidini Fluor 488 Reagent (ab176753, Abcam, Cambridge, MA), PrimeScript RT Master Mix (#RR036A), and TB Green Premix Ex Taq (RR420A) were from Takara Bio Inc. (Shiga Prefecture, Japan). Bone resorption ability was tested using the Corning Osteo Assay Surface Multiple Well Plate (Corning, Inc., Corning, NY, United States).

### Bone Marrow–Derived Macrophage Isolation

As reported previously, BMMs were obtained and cultured (Qu et al., 2014; Wang et al., 2019; Sun et al., 2020). In this present study, BMMs were isolated from the femurs and tibias of the 4-/6-week-old mice. After euthanizing with isoflurane, the muscle tissue of the femurs and tibias of the mice was removed in clean bench, followed by immersion in 75% ethyl alcohol for 10 min and phosphate-buffered saline (PBS) with 1% P/S for another 5 min. The bilateral ends of the femurs and tibias were then cut. Next, the whole bone marrow fluid was flushed using a sterile 5-ml syringe with complete α-MEM supplemented with 10% FBS, 1% P/S, and 30 ng/ml M-CSF, and then these isolated plugs were dispelled into single cell and seed in a T-75 cm^2^ flask for proliferation in a 37°C incubator with humidified atmosphere of 95% air and 5% CO_2_ for 3 days. Under the stimulation of M-CSF, only BMMs could adhere and survive. Therefore, when changing the medium, residual stromal cells would be washed and eliminated, and adherent cells on dish bottoms were classified as BMMs. Adherent cells that were grown to 95% confluence would be washed with PBS three times and trypsinized (EDTA^+^) for 30 min to harvest BMMs, and were either passaged or used for the following experiments.

### Cell Viability Assay

The cytotoxicity of CS-6 on BMMs was determined by the CCK-8 assay kit. Briefly, the BMMs were seeded and cultured in 96-well plates, with a density of 4 × 10^3^ cells per well (30 ng/ml M-CSF) overnight. Then, cells were added with indicated concentration of CS-6 (0, 1, 5, 10, 20, 40, 60, 80, 100, 150, and 200 nM) for 24, 48, or 96 h, respectively. Afterward, 100 μl test solution (90 μl α-MEM containing with 10 μl CCK-8 buffer) was added to each well. All plates were incubated at 37°C for 2–3 h. The absorbance was then measured at a wavelength of 450 nm on an ELX800 absorbance microplate reader (Bio-Tek, United States).

### OC Differentiation Assay *In Vitro*

BMMs were treated with complete α-MEM culture medium containing 10% FBS, 30 ng/ml M-CSF, and 1% P/S with a density of 8,000–10,000 cells per well in 96-well plates. For early intervention, RANKL (50 ng/ml) without or with CS-6 with various concentrations (5, 50, 100, and 150 nM) was added at the seconded day for 5–7 days, whereas for late intervention, BMMs were treated with RANKL (50 ng/ml) 3 days prior to adding CS-6 with indicated concentrations, and cultured for another 3–4 days. The medium containing M-CSF, RANKL, or CS-6 was replaced every 2 days. TRAP staining was described previously (Feng et al., 2017). When the fused cells could be observed in light microscope (Nikon, Tokyo, Japan), BMM cells were fixed with paraformaldehyde (4% PFA) for 15–20 min and then stained in TRAP working solution for 30 min at 37°C: A solution contained 50 μl Fast Garnet GBC Base Solution and 50 μl sodium nitrite solution (mixing for 30 s and letting it rest at room temperature for about 2 min); B solution comprised 200 μl acetate solution, 50 μl naphthol AS-BI phosphate as a substrate, and 100 μl tartrate solution, dissolving in 4.5 ml ddH_2_O; TRAP-stained cells were observed and imaged under light microscopy (Nikon, Tokyo, Japan), and the number and size of TRAP^+^ OCs with three or more nuclei were evaluated using Image J software (National Institutes of Health, Bethesda, MD).

### Podosome F-Actin Belt Immunofluorescence

When the fused cells could be observed in light microscope (Nikon, Tokyo, Japan), the cells were performed by F-actin ring staining. The method was described previously (Sun et al., 2020). Fused OCs were first fixed in 4% paraformaldehyde for 10–15 min, permeabilized using 0.15% Triton X-100 for another 5 min, washed three times with PBS, and then incubated with Phalloidin-iFluor 488 Reagent (CA1610, Solarbio, Shanghai, China) in 1% BSA-PBST for 30 min at room temperature. In addition, nuclei were counterstained with DAPI staining for additional 5 min. Fluorescence images of podosome F-actin belt were acquired using fluorescence microscopy and quantified by Image J software (National Institutes of Health, Bethesda, MD).

### Bone Resorption Assay

The bone resorption assay was described previously and conducted using the Corning osteo assay surface multiple well plate (Corning, Inc., Corning, NY, United States) (Feng et al., 2017; Lee et al., 2019). Briefly, BMMs were seeded and cultured in 96-well plates (8 × 10^3^ per well) in α-MEM supplemented with or without CS-6 as indicated above for 10–14 days. After elimination of OCs, the plates were stained with von Kossa (GP1054, Servicebio, Wuhan, China) in order to make the bony resorption pits more clear than normal surface coating. The percentage of the resorbed areas in three random resorption sites was measured using Image J software (National Institutes of Health, Bethesda, MD).

### RNA Isolation, RT-PCR, and Real-Time Quantitative PCR

BMM cells were seeded in 12-well plates, with a density of 2 × 10^5^ cells per well, and intervened in similar methods to those described in the section of OC differentiation assay *in vitro*.

First, the total cellular RNA was isolated from the BMMs using fast RNA extraction kit (Magen, Inc. Guangzhou, China), complying with the manufacturer’s instructions. The acquired cDNA was then reversed from 1 to 2 μg of the total RNA using reverse transcription kit (TaKaRa Biotechnology, Otsu, Japan). Subsequently, the mRNA expression levels were quantified by qRT-PCR with the SYBR Premix Ex Tag kit (TaKaRa, Biotechnology, Otsu, Japan) using the real-time PCR system (Applied Biosystems, Foster City, CA, United States). The reaction conditions were as follows: 40 cycles of denaturation at 95°C for 10 s and amplification at 60°C for 30 s. All reactions were run for three times, and the gene expression levels were normalized to those of GAPDH. The primer sequences used for qRT-qPCR analysis are shown in Supplementary Table S1.

### Western Blot Analysis

The method was similar to that reported by Sun et al. (Sun et al., 2020). To investigate the effect of CS-6 on RANKL-induced signaling cascades, BMMs were seeded with a density of 5 × 10^5^ cells/well and cultured with α-MEM (M-CSF, 30 ng/ml) for 24 h. Then, the BMMs were treated with a serum-free α-MEM (without M-CSF) for 1 h prior to being stimulated without or with CS-6 of indicated concentrations (0, 5, 50, 100, and 150 nM) for 3–4 h, and then cells were stimulated with 50 ng/ml RANKL for additional 30 min. In terms of the effect of CS-6 on the protein expression of c-Fos, NFATc1, MMP9, and TRAF6 during OC differentiation, BMMs were plated with complete α-MEM containing M-CSF (30 ng/ml) for 24 h, and RANKL (50 ng/ml) without or with CS-6 with various concentrations (0, 5, 50, and 100 nM) was added at the seconded day for another 3 days, whereas for late intervention, CS-6 with indicated concentrations was added at the third day following RANKL (50 ng/ml) for another 3 days.

For protein extraction, differently treated BMMs were completely lyzed in ice-cold RIPA and cleared by centrifugation, and protein concentration was quantified using the BCA Protein Assay kit (Beyotime Institute of Technology, Shanghai, China). A total of 30–40 μg protein/lane were added onto 10% sodium dodecyl sulfate polyacrylamide gel and separated by electrophoresis (80 V, 120 min). Then, the separated protein bands were transferred onto a polyvinylidene fluoride membrane (EMD Millipore, Billerica, MA, United States) for 100 min (100 V). Subsequently, blocking was conducted by dissolving 5% nonfat milk in Tris-buffered saline-Tween for at least 2 h at room temperature (Invitrogen, San Diego, CA, United States). Next, the PVDF membranes were washed with TBST (Tris-buffered saline with 0.1% Tween 20) three times (5 min per time). The membranes were then cut according to protein marker and incubated with primary antibodies above at 4°C temperature overnight. Subsequently, the membranes were then incubated with the indicated secondary antibodies (goat against rabbit or mouse, 1:5,000) for additional 2 h at room temperature. Finally, the target protein bands were visualized using a Tanon Imaging System (version 5,200, Tanon Science and Technology Co., Ltd., High-tech Park, Shanghai, China) and quantified using Image J software (National Institutes of Health, Bethesda, MD).

### Nuclear Translocation of the NF-kB p65 Assay

BMMs were seeded and cultured with complete α-MEM containing M-CSF (30 ng/ml) in 6-well plates for 24 h. Then, the BMMs were treated with a serum-free α-MEM (without M-CSF) for 1 h prior to being stimulated without or with CS-6 of indicated concentrations (100 nM) for 3–4 h. Then, cells were stimulated with or without RANKL (50 ng/ml) for 30 min. Next, the BMMs were then fixed using 4% paraformaldehyde for 15–20 min and permeabilized in 0.1% Triton X-100 for 30 min at RT. Subsequently, cells were incubated with 5% nonfat milk in PBST overnight. After incubation with p65 antibody (1:100 in 1% BSA) for 12 h at 4°C, cells were incubated with FITC-conjugated goat anti-rabbit IgG (Servicebio, Wuhan, China) for 1 h and then stained with DAPI (Sigma; St. Louis, MO, United States) for 10 min in the dark at RT. Cells were washed three times with PBS and imaged using a laser scanning confocal microscope.

### Establishment of Ovariectomy (OVX)-Induced Bone Loss Model *In Vivo*

In order to minimize the damage to mice, we established the ovariectomy (OVX)-induced bone loss model *via* a dorsal approach, similar to previous report (Chen et al., 2019). Briefly, the mice were kept in a prone position. After being anesthetized with inhalational isoflurane (2.5%), the mice were placed prone on a warming pad with the temperature of 37°C. Subsequently, a single longitudinal incision at midline dorsal skin (l cm length) was made. The ovarian fat pad was then gently dissected using ophthalmic forceps to expose the ovaries. Bilateral ovaries were then resected after tubal ligation, and the fat pad was placed into its previous position. Finally, the incision was closed layer by layer. The sham control group underwent the same procedure, except reserving the ovaries. The whole procedure was carried out in a specific pathogen-free environment.

Seven days after operation, the administration of DMSO, β-estradiol, or CS-6 would be performed every 2 days over the entire period of experiment of 4 weeks. Twenty-five 8-week-old female C57BL/6 mice with a mean weight of 20 g were purchased from Slack Institution (Shanghai, China) and randomly allocated into five groups (*n* = 5), including the sham group (mock operation with DMSO injection), OVX group (OVX with DMSO injection), positive group (OVX with β-estradiol injection, 5 mg/kg), low-dose group (OVX with low-dose CS-6, 0.5 mg/kg), and high-dose group (OVX with high-dose CS-6, 2.5 mg/kg). At the end of the experiment, mice were euthanatized with isoflurane, and then the femurs and uterus were collected fixed in 4% PFA for 2 days and changed to 70% alcohol, as well as the arterial blood samples for the following experiments.

### Microcomputed Tomography and Histological Analysis

The analysis of Bone morphometry and bone histomorphometry was performed according to the guidelines and nomenclature definitions of ASBMR (Bouxsein et al., 2010). The three-dimensional (3D) structure of the femurs was evaluated using micro-CT scanner (Skyscan1176, United States Bruker Siemens Inveon, Eschborn, Germany) with the following parameters: voltage of 50 kV, current of 450–500 μA, and isotropic resolution of 9 μm. A 0.5-mm thick aluminum filter was used for beam-hardening reduction. Quantitative morphometric analyses were carried out with a square region of interest (ROI) 50 cross-sectional planes (0.5 mm) below the femur growth plate for analysis of 200 cross-sectional planes in height (2 mm) for trabecular bone. The obtained images were built with NRecon software (Bruker microCT, Kontich, Belgium) using the following parameter settings: ring artifact correction, 8; smoothing, two; and beam hardening correction, 30–40%. Bone parameters analyzed for femur bone samples included bone volume/total volume (BV/TV), bone surface to total volume ratio (BS/TV, mm^−1^), trabecular thickness (Tb.Th, mm), trabecular spacing (Tb.Sp, mm), and trabecular number (Tb.N, mm^−1^), and the trabecular bone mineral density (BMD) was analyzed, within the selected metaphyseal region. Quantitative morphometric analyses were carried out with a square region of interest (ROI) 500 cross-sectional planes (5 mm) below the femur growth plate for analysis of 50 cross-sectional planes in height (0.5 mm) for cortical bone. For analysis of cortical bone morphology parameters, we included the total cross-sectional area inside the periosteal envelope (Tt.Ar, mm^2^), cortical bone area (Ct.Ar, mm^2^), cortical area fraction (Ct.Ar/Tt. Ar, %), and average cortical thickness (Ct.Th, mm).

After μCT analysis, the bone samples were further decalcified in EDTA solution (10%) for another 3–4 weeks, and then embedded in paraffin blocks for following sectioning. Sections were then stained with hematoxylin eosin or TRAP. The area of trabecular bone and the number of OCs were quantified using the Image J software (National Institutes of Health, Bethesda, MD) (Chen et al., 2019; Sun, et al., 2020).

### ELISA

Serum levels of IL-1β, TNF-α, RANKL, TRACP5b (tartrate-resistant acid phosphatase 5b), CTX-1(C-telopeptide of type I collagen), and osteoprotegerin (OPG) were measured with mouse ELISA kits under the guidance of the manufacturer’s protocols (Westang, Shanghai, China).

### Statistical Analysis

All quantified data in this study are presented as mean ± standard deviation.

Statistical analyses were performed using GraphPad Prism 8 (GraphPad Software Inc.; La Jolla, CA) for Windows adopting one-way analysis of variance (ANOVA), followed by the Student–Neuman–Keuls *post hoc* test to make comparisons between groups. *p* values less than 0.05 or otherwise indicated a statistical difference.

## Results

### CS-6 Suppresses RANKL-Induced Osteoclastogenesis *In Vitro*

**FIGURE 1:**
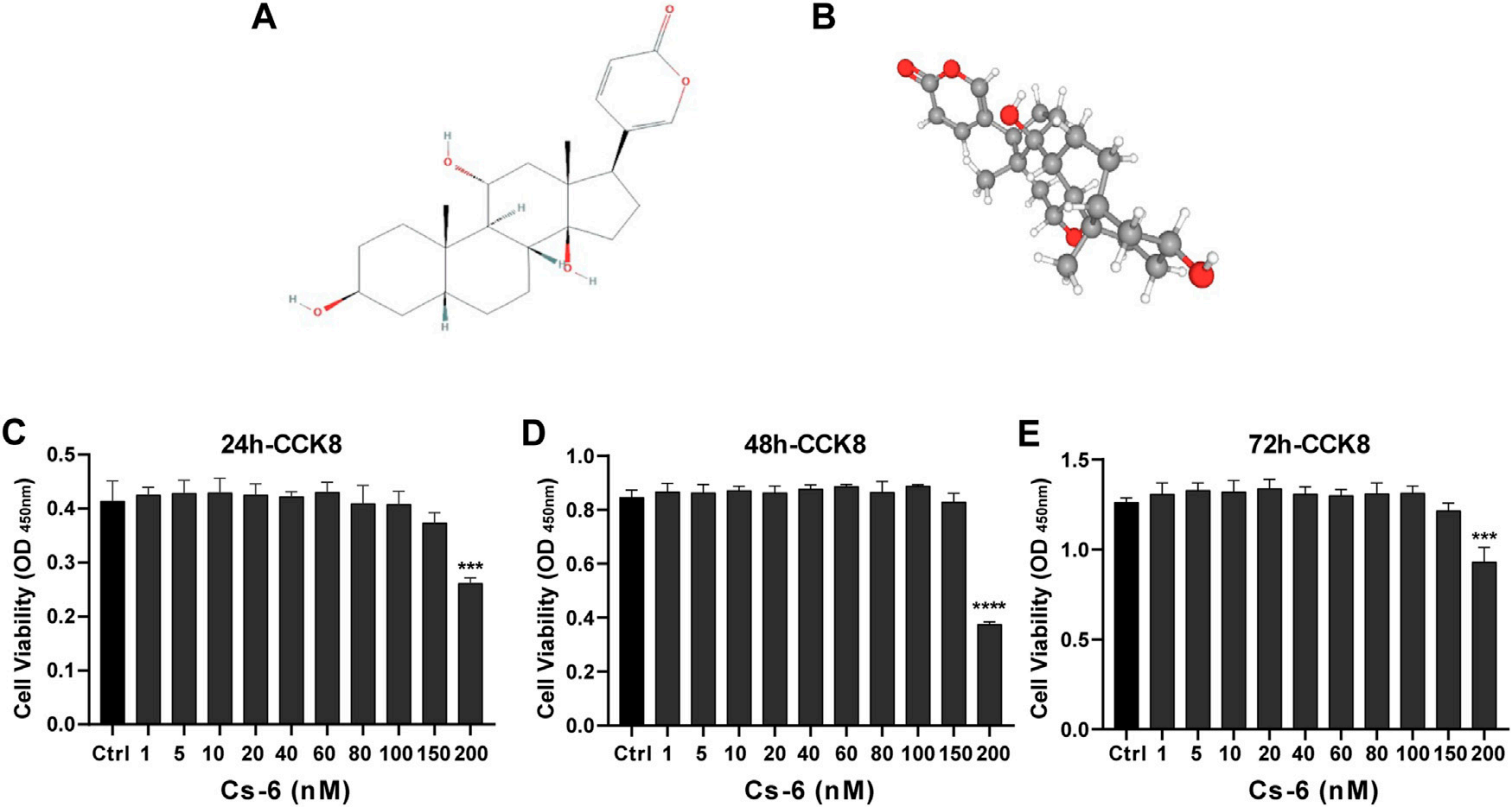
Chemical structure and cytotoxicity of gamabufotalin (CS-6) on osteoclast precursor cells (bone marrow–derived monocyte–macrophage (BMM) lineage). **(A, B)** Chemical structure of CS-6. **(C–E)** Cell viability of BMMs as evaluated by CCK-8 assay following treatment with or without indicated concentrations of CS-6 at the time point of **(C)** 24, **(D)** 48, and **(E)** 96 h, respectively. Values were presented as the mean ± standard deviation (*n* = 5); *p < 0.05, **p < 0.01, ***p < 0.001, ****p < 0.0001.

**FIGURE 2:**
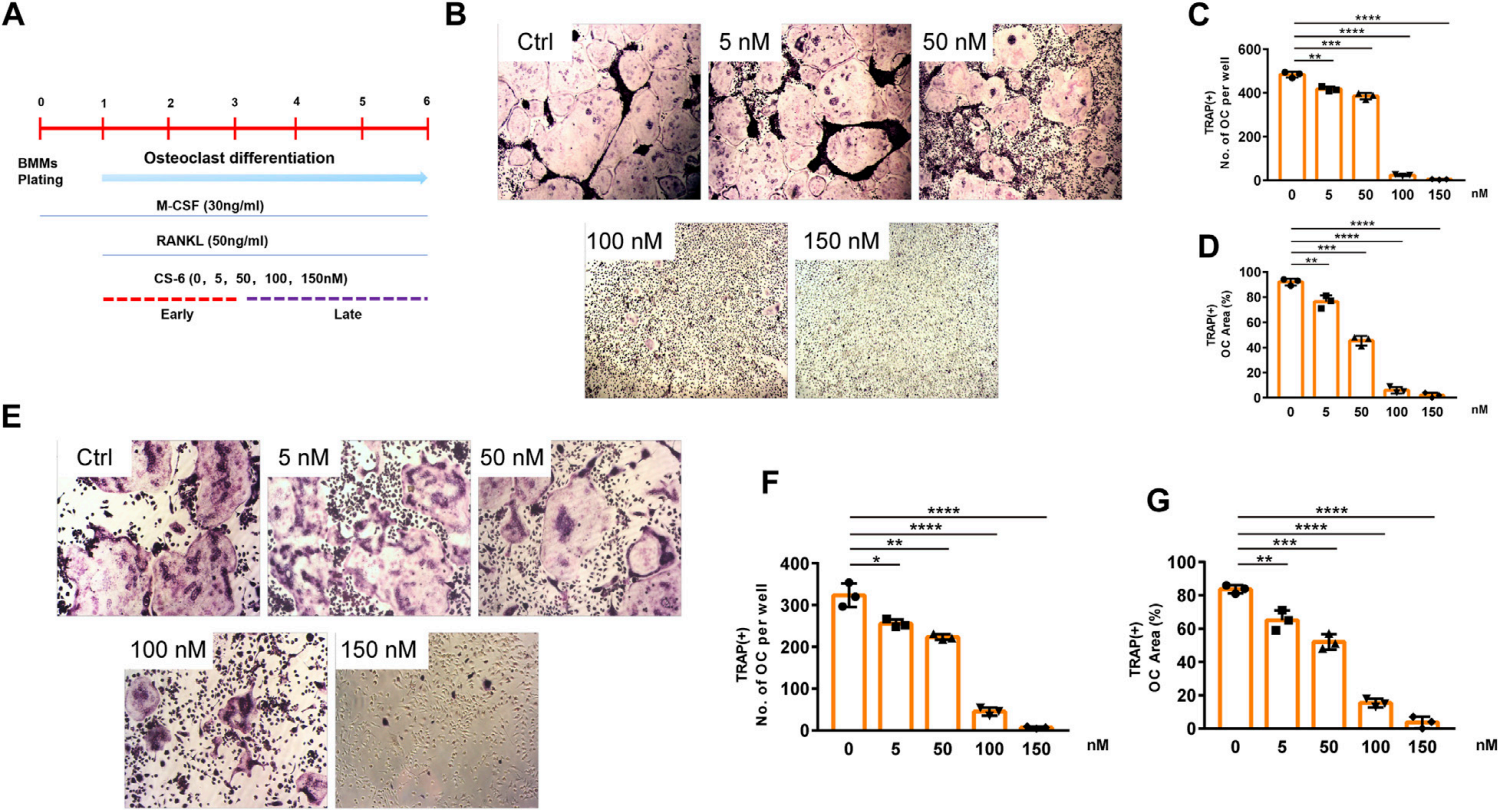
CS-6 inhibited RANKL-induced OC differentiation *in vitro* in both the early and late stages of osteoclastogenesis. **(A)** The illustration of experimental design regarding early and late stimulation by CS-6. **(B)** Representative images of TRAP staining–positive OCs stimulated by RANKL (50 ng/ml) with indicated concentrations of CS-6 for 5–7 days. The **(C)** number and cell spread area **(D)** of TRAP^+^ multinucleated OCs were shown as graphs. **(E)** Representative images of TRAP staining–positive OCs stimulated with RANKL (50 ng/ml) for 3 days prior to indicated concentrations of CS-6. The **(F)** number and cell spread area **(G)** of TRAP^+^ multinucleated OCs were presented as graphs. Values were shown as the mean ± standard deviation (*n* = 3); *p < 0.05, **p < 0.01, ***p < 0.001, ****p < 0.0001.

The chemical structure of CS-6 is presented in Figure 1 (Figures 1A,B). To investigate the cytotoxic effect of CS-6 on BMMs, the cell viability was examined by CCK8 viability assay, and we found that CS-6 below 200 nM exhibited no dramatic damage to BMMs (Figures 1C–E). Based on the results above, we then explored the effect of CS-6 on the formation of functional OCs induced by RANKL *in vitro*. The experimental design is shown in Figure 2 (Figure 2A). BMMs were incubated with M-CSF (30 ng/ml) for 24 h prior to being treated with RANKL (50 ng/ml) with or without CS-6 at concentrations of 5, 50, 100, and 150 nM for 6–7 days. As shown in Figure 2, CS-6 reduced the number of TRAP^+^ multinucleated cells in a dose-dependent manner (Figures 2B–D). Furthermore, treatment with CS-6 also evidently reduced the size of OCs, indicating the potential inhibitory effect of CS-6 on precursor cell fusion and/or cytoskeletal alterations.

Further, to identify the exact stage of osteoclastogenesis that CS-6 affected OC differentiation, BMMs were treated with RANKL (50 ng/ml) three days prior to adding CS-6 at concentrations of 5, 50, 100, and 150 nM for another 3 days. As shown in Figure 2, CS-6 could also exert significant inhibitory effects on OC formation at late stage (Figures 2E–G).

These results above indicated that CS-6 could inhibit the formation of functional OCs induced by RANKL in both early and late phase of OCs development.

### CS-6 Suppresses the Formation of F-Actin Ring and Bone Resorption Activity of OCs

**FIGURE 3:**
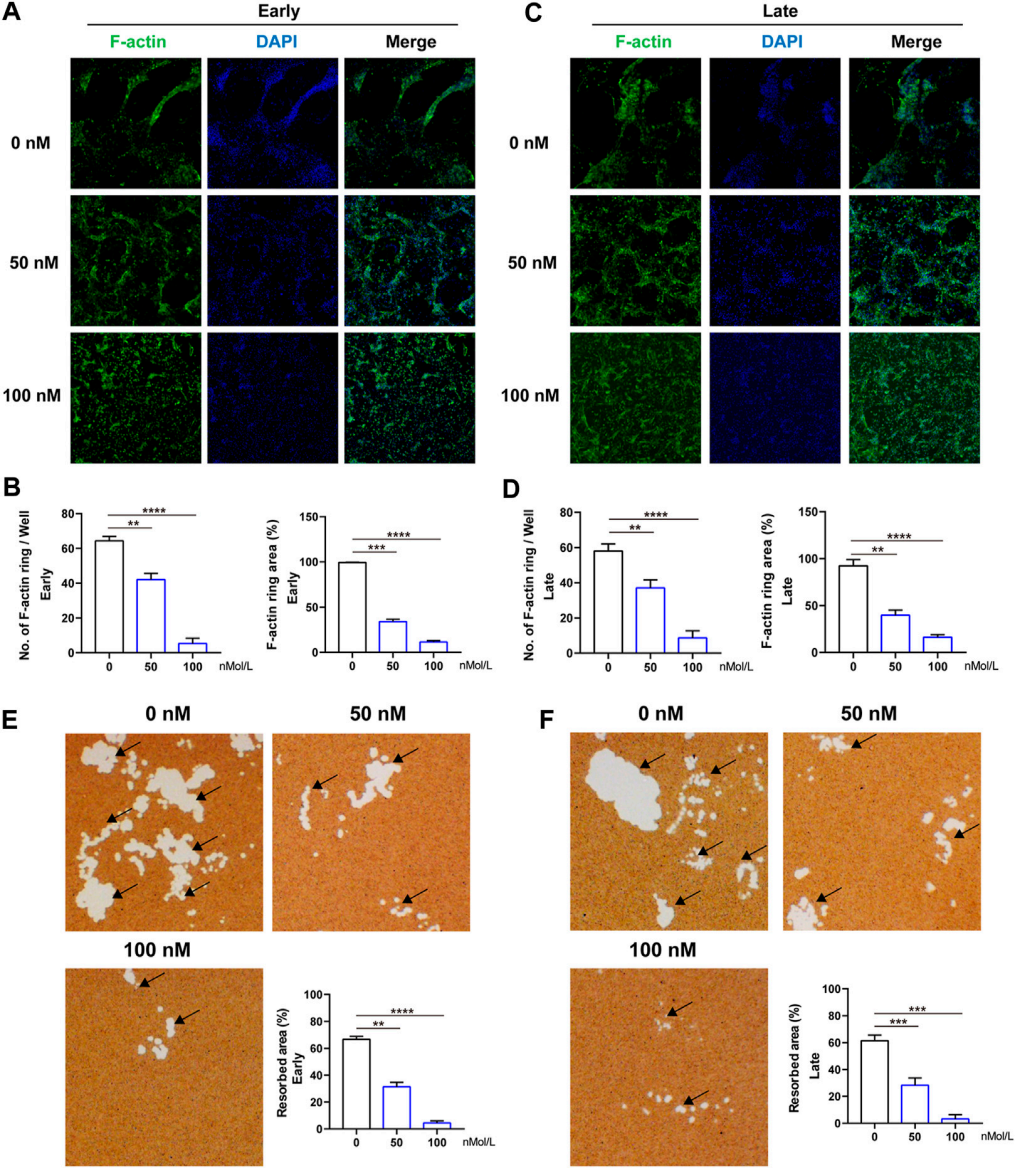
CS-6 suppresses the formation of F-actin ring and bone resorption of OCs. **(A)** Representative images of F-actin (Green) formed by OCs stimulated by RANKL (50 ng/ml) with indicated concentrations of CS-6 (100 nM) for 5–7 days. **(B)** The average number and area of F-actin ring. **(C)** Representative fluorescence images of actin-stained BMM-derived OCs stimulated with RANKL (50 ng/ml) for 3 days prior to indicated concentrations of CS-6 (100 nM). **(D)** The average number and area of F-actin ring. **(E)** Representative images of bone resorption pits (white area marked by black arrow) by mature OCs stimulated by RANKL (50 ng/ml) with indicated concentrations of CS-6 (100 nM) for 5–7 days and quantified graph. **(F)** Representative images of bone resorption pits (white area marked by black arrow) by mature OCs stimulated by RANKL (50 ng/ml) for 3 days prior to indicated concentrations of CS-6 (100 nM) and quantified graph. Values presented as the mean ± standard deviation (*n* = 3). *p < 0.05, **p < 0.01, ***p < 0.001, ****p < 0.0001.

The formation of F-actin belt surrounding the individual OC is the most typical feature of mature multinucleated OCs during osteoclastogenesis, and is indispensable for subsequent bone-resorptive activity. Therefore, FITC–phalloidin staining was used to evaluate the F-actin ring formation first. As indicated in Figure 3, almost all the OCs induced by RANKL have typical F-actin ring (Figure 3A), whereas after treated by CS-6 (50 and 100 nM), the number and size of the F-actin ring were dramatically decreased in both the early and late stages of osteoclastogenesis, consistent with the TRAP-stained results above (Figures 3A–D). As a result, CS-6 could suppress the formation of F-actin ring of mature OCs in both the early and late stages of OC differentiation.

Meanwhile, an intact F-actin ring is an essential pre-requisite for OC bone-resorptive activity. Thus, we then explored whether CS-6 could impair the bone-resorptive function of mature OCs; Corning osteo assay surface multiple well plate was used to mimic the bone tissue. BMMs were cultured on the plate and induced with M-CSF (30 ng/ml) and RANKL (50 ng/ml) in the absence or presence of CS-6 with indicated concentrations. As shown in Figure 3, CS-6 could reduce the bone-resorptive area in a dose-dependent manner during both the early and late periods of osteoclastogenesis compared with groups with only RANKL stimulation which showed obvious clearing of the hydroxyapatite substrate (Figure 3, white regions E and F). In addition, approximately 40 and 50% reduction was seen in OCs treated with 50 and 100 nM CS-6, respectively (Figures 3E,F).

Collectively, CS-6 could inhibit the F-actin ring construction as well as the bone-resorptive activity of OCs.

### CS-6 Suppresses RANKL-Mediated Marker Gene Expression in Osteoclastogenesis

**FIGURE 4:**
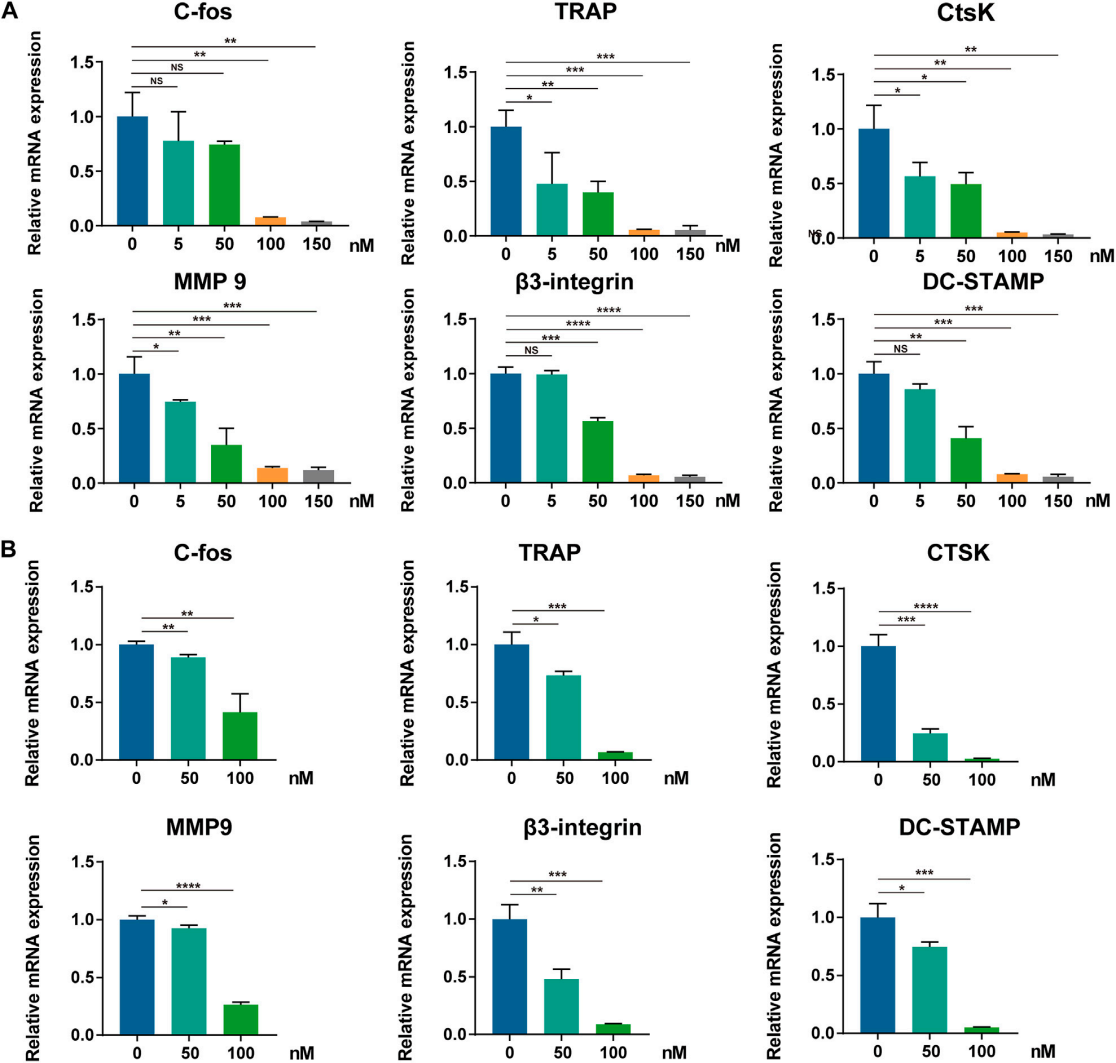
CS-6 suppresses RANKL-mediated marker genes expression in osteoclastogenesis. **(A, B)** The relative mRNA expression of OC-related genes (c-Fos, TRAP, CTSK, MMP-9, DC-STAMP, and β3-integrin) following CS-6 treatment was measured by qRT-PCR in the early **(A)** and late **(B)** stages of stimulation. Values presented as the mean ± standard deviation. *p < 0.05, **p < 0.01, ***p < 0.001, ****p < 0.0001.

To further determine the molecular mechanism underlying the suppressive effect of CS-6 on OCs, the mRNA expression of OC-related marker genes was analyzed first using qRT-PCR. Following treatment with CS-6 with indicated concentrations, the mRNA expression of OC marker genes was markedly down-regulated during osteoclastogenesis, including c-Fos, TRAP, CTSK, MMP9, β3-Integrin, and DC-STAMP, in a dose-dependent manner (Figure 4A). Additionally, when BMMs were treated with M-CSF (30 ng/ml) and RANKL (50 ng/ml) 3 days prior to CS-6 for another 3 days, the mRNA expression of OC marker genes was also suppressed (Figure 4B). Therefore, CS-6 impaired the formation of functional OCs by inhibiting RANKL-induced OC gene mRNA expression.

### CS-6 Attenuates RANKL-Induced Activation of NF-κB and ERK/MAPK Signaling Cascades, and Downstream Transcriptional Activity of c-Fos and NFATc1

**FIGURE 5:**
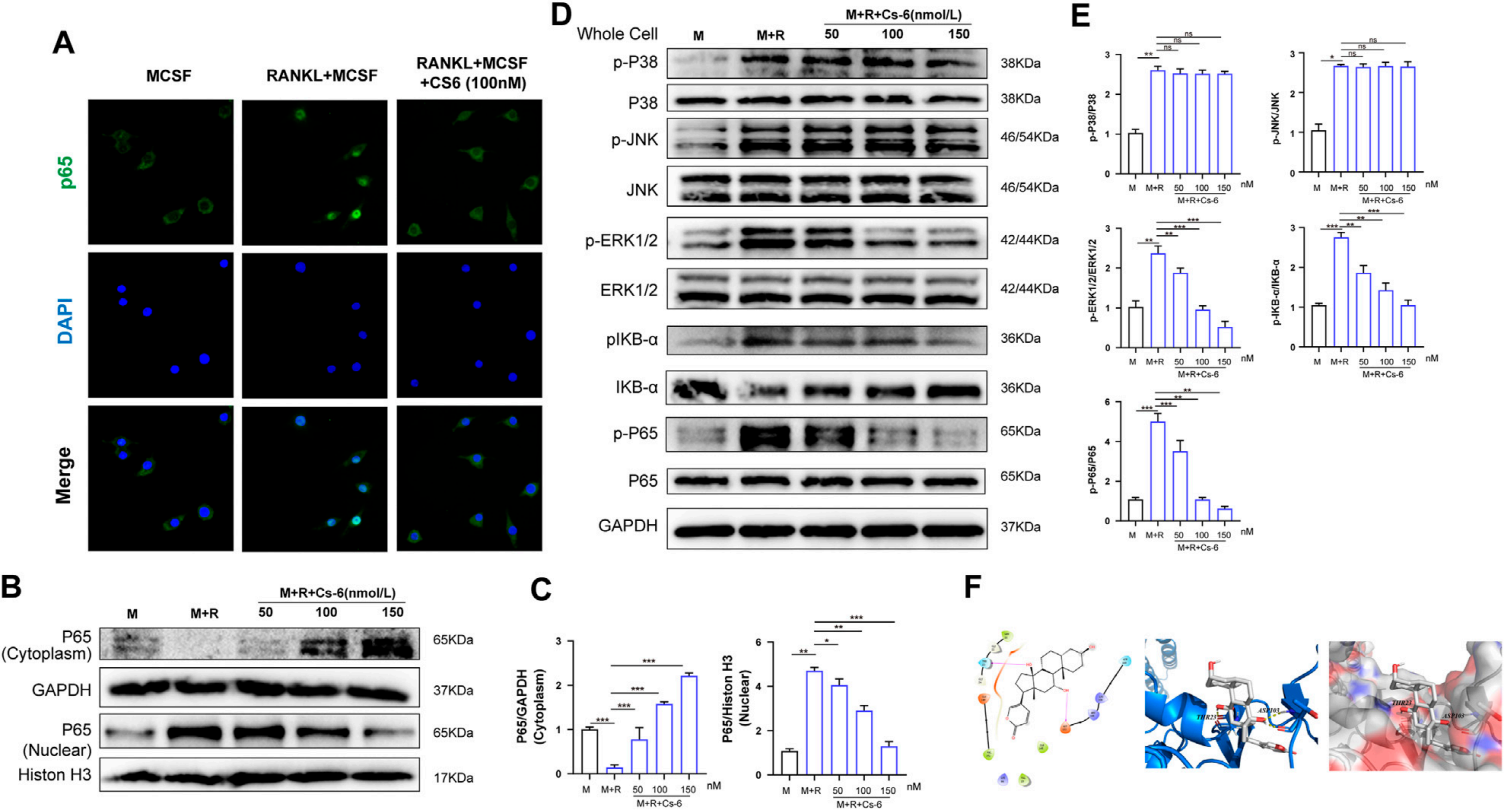
CS-6 attenuated RANKL-induced activation of NF-κB and ERK/MAPK signaling pathways. **(A)** Representative immunofluorescence images regarding the distribution of p65 in BMMs treated by RANKL (50 ng/ml) with or without CS-6 (100 nM) compared to the control group (30 ng/ml MCSF only). **(B, C)** The protein amount of p65 in cytoplasm and the nucleus was measured by Western blot and quantified, respectively. **(D)** BMM cells were pretreated with CS-6 (0, 50, 100, and 150 nM) for 3 h prior to stimulation with RANKL for additional 30 min. The total protein amount of the whole cell was determined by Western blot. The key proteins of NF-κB and MAPK pathways were shown. **(E)** The expression of total proteins was quantified by graphs. **(F)** **(Left)** The predicted best binding manner of CS6 in the ATP binding site of IKKβ generated with docking; hydrogen bonds were shown as purple lines. **(Middle)** Co-crystal structure of the interactions between CS6 and IKKβ; hydrogen bonds were displayed as red lines, and the participating amino acid residues (Thr23 and Asp103) were marked. **(Right)** MOLCAD representation of the molecular lipophilic potential surface upon the bioactive pose of CS6 in the ATP binding site of IKKβ. The blue denoted the hydrophilic, red for the lipophilic, and gray denoted neutral moiety. Values were presented as the mean ± standard deviation (*n* = 3); *p < 0.05, **p < 0.01, ***p < 0.001, ****p < 0.0001.

Timely and well-organized activation of a series of OC differentiation–related signaling pathways induced by RANKL are crucial to OC differentiation. To identify the potential signaling pathways involved in OC formation after treatment with CS-6, we explored the early activation of two critical RANKL-initiated pathways, the NF-kB and MAPK. In terms of the early activation of the NF-kB signaling cascade, the early nucleus translocation of p65 after RANKL stimulation as determined by the immune-fluorescent staining suggested a decrease trend when CS-6 was added in BMMs (Figure 5A). In addition, we also examined the amount of p65 inside the BMM nucleus and found that the amount of p65 inside the nucleus was also decreased co-treated with CS-6 (Figures 5B,C). On the other hand, IκB kinase (IKK) complex is essential for the activation of NF-κB signaling, and its one unit, IκB-α, is a major upstream kinase and could result in the activation of the NF-κB signaling pathway. We then investigated whether CS-6 could exert influence on IKK activity with the aim to better understand the inhibitory effect of CS-6 on the activation of NF-kB, and found that the degradation of Ik-Bα as well as the phosphorylation of NF-kB and Ik-Bα was inhibited by CS-6 in a dose-dependent manner after RANKL stimulation for 30 min (Figures 5D,E). Hence, we made the hypothesis that CS-6 could affect the activation of IKK. To verify our hypothesis, molecular interaction assay was carried out to simulate the potential interactions between CS-6 and IKK, and the result indicated that CS-6 could well bind to the ATP binding site of IKKβ. Specifically, as shown in Figure 5F (left and middle), CS-6 could bind to the ATP binding pocket of the IKKβ kinase domain (Thr23 and Asp103) *via* two hydrogen bonds. In addition, the result of MOLCAD surface modeling indicated that CS-6 contained lactone ring and could spread into the deep hydrophobic region of the ATP-binding pocket of the molecular lipophilic potential surface of IKKβ (Figure 5F, Right). These results provided evidence for the inhibitory effect of CS-6 on the activation of the Nf-κB signaling pathway. Moreover, RANKL-induced phosphorylation of ERK was also suppressed by CS-6 (Figures 5D,E). However, co-treatment with CS-6 showed no obvious impact on the activation of p38 and JNK (Figures 5D,E).

**FIGURE 6:**
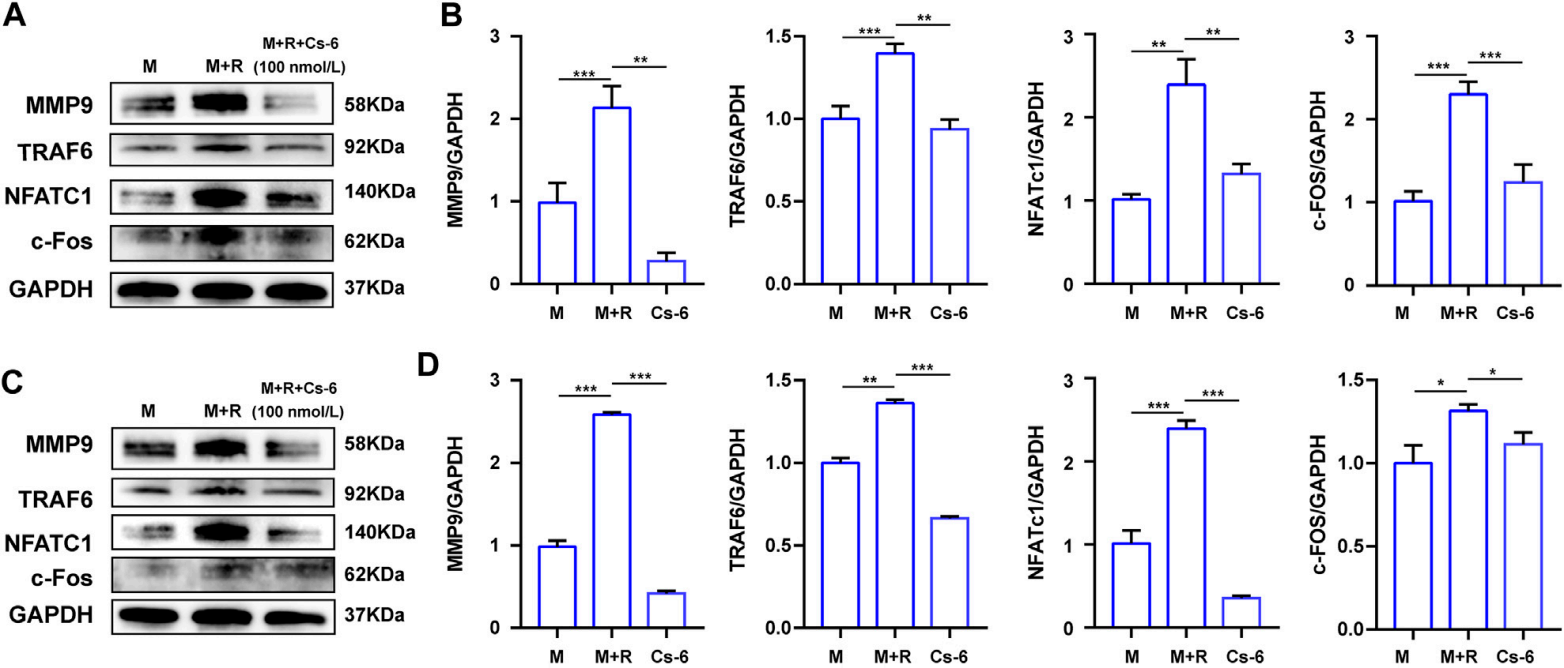
CS-6 suppressed the protein expression of the OC marker genes. **(A)** The results of the protein expression of OC-related genes including TRAF6, MMP-9, c-Fos, and NFATc1 in BMM cells stimulated with RANKL (50 ng/ml) with indicated concentrations of CS-6 (100 nM) for 5–7 days compared to the control group (30 ng/ml MCSF only). **(B)** The expression of related protein was quantified. **(C)** The results of the protein expression of OC-related genes including TRAF6, MMP-9, c-Fos, and NFATc1 in BMM cells stimulated with RANKL (50 ng/ml) for 3 days prior to indicated concentrations of CS-6 (100 nM) compared to the control group (30 ng/ml MCSF only). **(D)** The expression of related protein was quantified. Values were presented as the mean ± standard deviation (*n* = 3); *p < 0.05, **p < 0.01, ***p < 0.001, ****p < 0.0001.

NFATc1 and c-Fos are the two essential transcription agents required for BMM fusion and formation of functional OCs by transcriptionally regulating the expression of numerous OC-related genes. Therefore, we investigated the effect of CS-6 on the protein expression of NFATc1 and c-Fos on the third days, and the Western blot results demonstrated that the expression of NFATc1 and c-Fos was elevated by RANKL, whereas CS-6 counteracted the activated effect of NFATc1 and c-Fos induced by RANKL (Figures 6A,B). In terms of the late effect of CS-6 on the expression of NFATc1 and c-Fos, the results showed the increased expression of these two regulators by RANKL was also ameliorated by CS-6 (Figures 6C,D). Additionally, the expression of TRAF6 and OC-related marker, MMP9, induced by RANKL was also significantly downregulated when treated with CS-6 in both the early and late stages of osteoclastogenesis (Figures 6A–D).

Consequently, CS-6 definitely possesses suppressive effects on OC differentiation by attenuating RANKL-induced activation of NF-κB and ERK/MAPK signaling pathways, and downstream transcriptional activity of c-Fos and NFATc1.

### Validation of the Biological Effect of CS-6 on OC Bone Resorption in Systemic OVX-Induced Bone Loss in Mice Model

**FIGURE 7:**
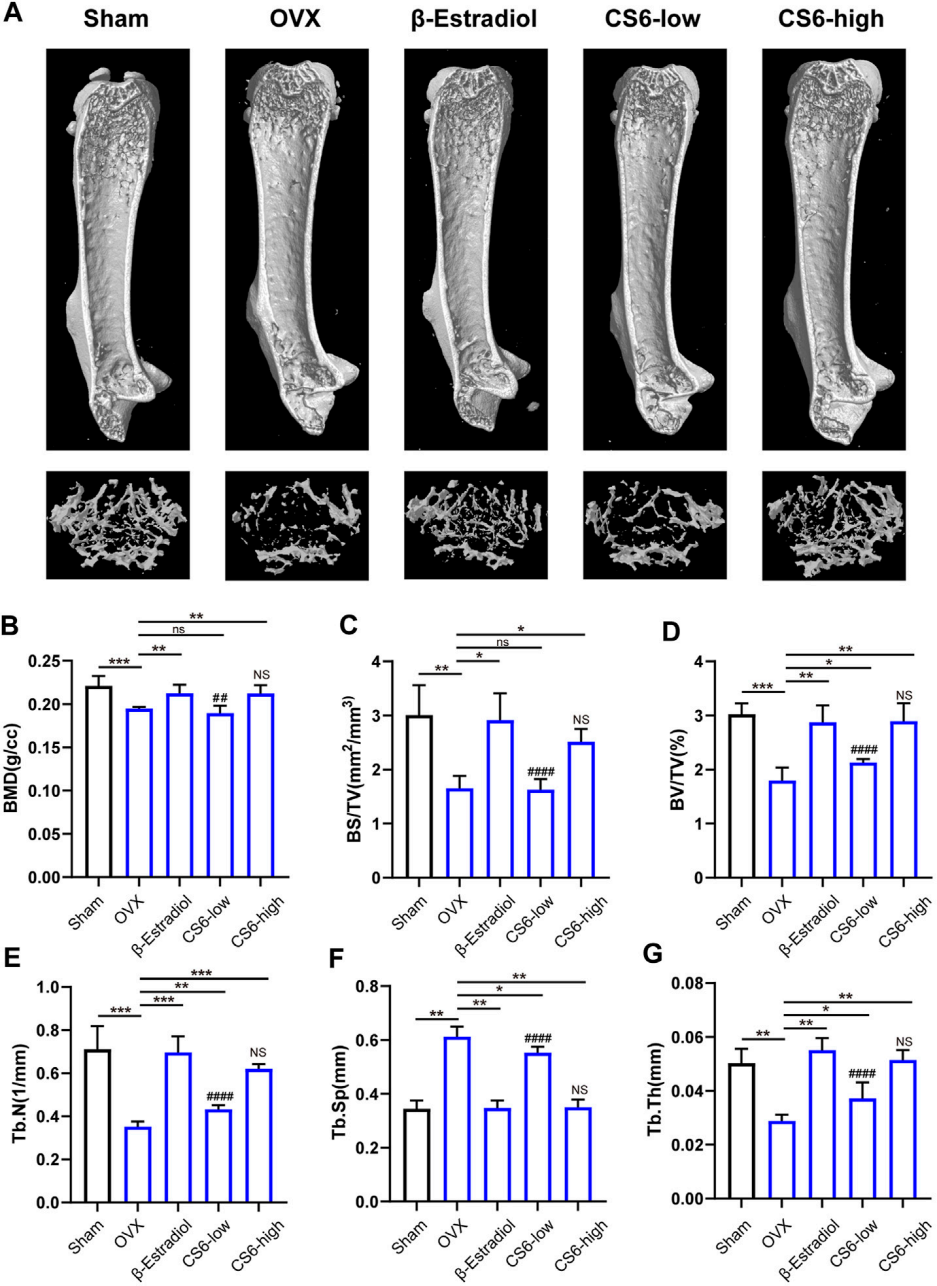
Validation of the biological effect of CS-6 on osteoclastic bone resorption in systemic OVX-induced bone loss *in vivo* indicated by microstructure analysis. **(A)** Representative three-dimensional images of μCT for mouse femur from the sham group (mock operation with DMSO injection), OVX group (OVX with DMSO injection), positive group (OVX with β-estradiol injection, 5 mg/kg), low-dose group (OVX with low-dose CS-6, 0.5 mg/kg), and high-dose group (OVX with high-dose CS-6, 2.5 mg/kg). **(B–E)** Quantitative analyses of bone structural parameters, including trabecular bone mineral density (BMD, g/cc), bone surface area/total volume (BS/TV; %), bone volume/total volume (BV/TV; mm^−1^), trabecular number (Tb.N; mm^−1^), trabecular spacing (Tb.Sp, mm), and trabecular thickness (Tb.Th, mm) within the selected metaphyseal region were shown in the charts. Values were presented as the mean ± standard deviation (*n* = 5); *p < 0.05, **p < 0.01, ***p < 0.001, ****p < 0.0001. # and NS indicated the comparison between the sham group and CS-6 treatment group, #*p* < 0.05, *p* < 0.01, *p* < 0.001, *p* < 0.0001.

Subsequently, we examined whether the encouraging effect of CS-6 on OC formation and bone resorption *in vitro* can be reproduced *in vivo*. Previous study has suggested that in postmenopausal osteoporosis, the number and activity of osteoclasts will be enhanced with the decrease of the level of estrogen, and lead to bone homeostasis imbalance and inflammatory bone loss (Sapir-Koren and Livshits, 2017). Therefore, the OVX mice model was established to verify the effect of CS-6. The microstructure of femur bone trabecula was determined by μCT. As shown in Figure 7, the obtained results demonstrated an improvement in trabecular bone mass following CS-6 co-treatment. The results of BMD, BV/TV, Tb.N, Tb.Th, Tb. Sp, and BS/BV suggested that OVX mice exhibited evident loss of trabecular bone, whereas this was reversed by β-estradiol or CS-6 administration (Figures 7A–G). Surprisingly, similar protective effects were seen between the OVX group with β-estradiol injected and OVX group with high-dose CS6 injected (2.5 mg/kg bodyweight; Figures 7A–G). Notably, the comparison of results between the CS-6 treatment groups and the sham control group also suggested that OVX mice with high-dose CS-6 showed similar μCT parameters (all *p* > 0.05). The results above indicated that these parameters could be returned to levels of the sham control group by the high dose of CS-6 administration. Interestingly, we also found that mice treated with high-dose CS-6 showed similar but not equivalent effect to β-estradiol in terms of the decreased body weight and increased ratio of uterus weight/body weight in OVX mice administrated with β-estradiol, or CS-6 compared OVX mice with DMSO only (Supplementary Figures S1A–C).

**FIGURE 8:**
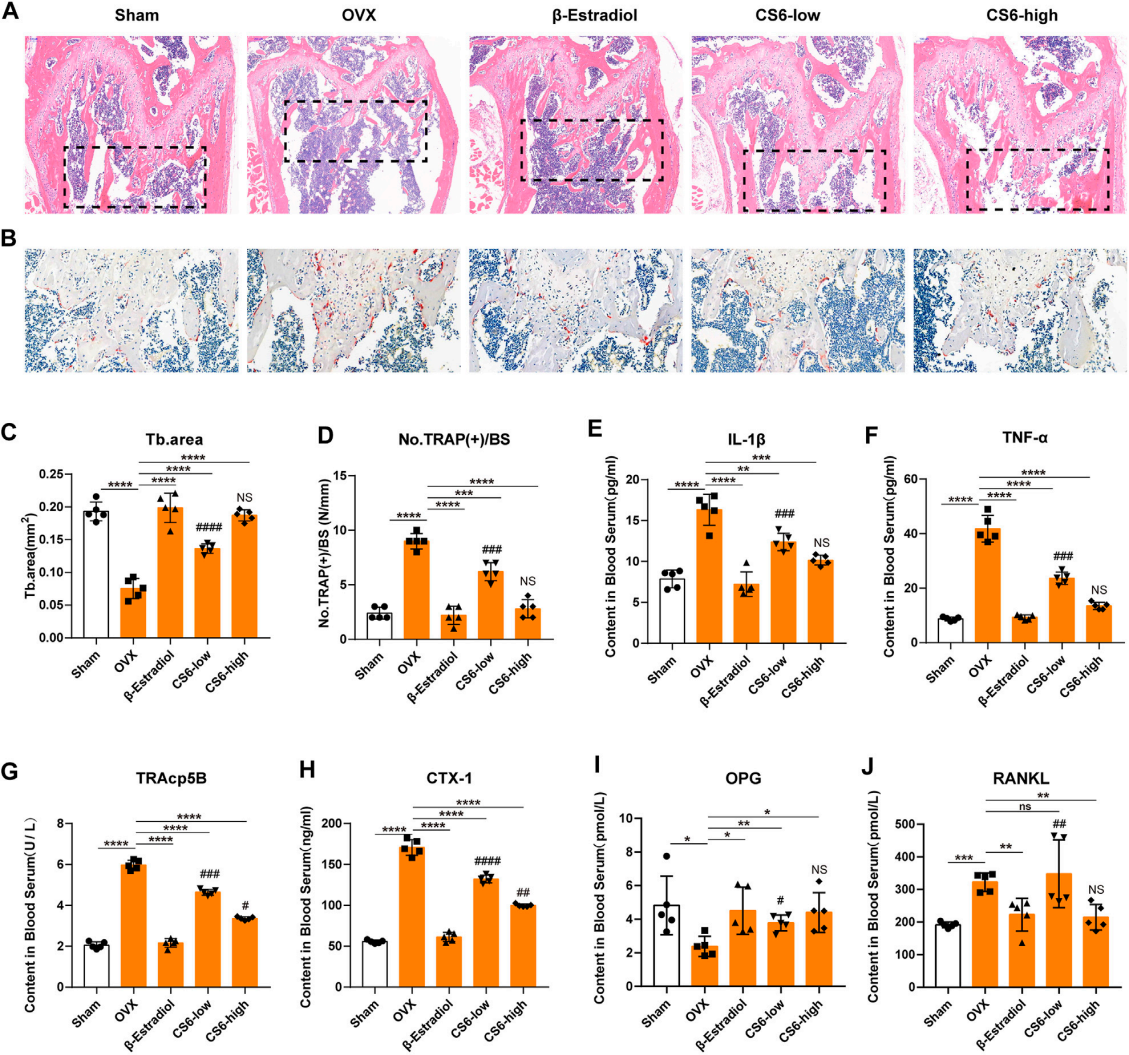
Validation of the biological effect of CS-6 on osteoclastic bone resorption in systemic OVX-induced bone loss *in vivo* indicated by histological analysis. **(A)** Representative images of H&E staining within the selected metaphyseal region from the sham group (mock operation with DMSO injection), OVX group (OVX with DMSO injection), positive group (OVX with β-estradiol injection, 5 mg/kg), low-dose group (OVX with low-dose CS-6, 0.5 mg/kg), and high-dose group (OVX with high-dose CS-6, 2.5 mg/kg). **(B)** Representative images of TRAO staining within the selected metaphyseal region from the sham group (mock operation with DMSO injection), OVX group (OVX with DMSO injection), positive group (OVX with β-estradiol injection, 5 mg/kg), low-dose group (OVX with low-dose CS-6, 0.5 mg/kg), and high-dose group (OVX with high-dose CS-6, 2.5 mg/kg). **(C)** The area of the trabecular bone was quantified. **(D)** The number of TRAP^+^ cells per bone surface was quantified. **(E–J)** The serum level of pro-inflammatory cytokines and OC-related markers was presented. Values were presented as the mean ± standard deviation (*n* = 5); *p < 0.05, **p < 0.01, ***p < 0.001, ****p < 0.0001. # and NS indicated the comparison between the sham group and CS-6 treatment group, #*p* < 0.05, ^##^ *p* < 0.01, ^###^ *p* < 0.001, ^####^ *p* < 0.0001.

As to histological analysis, H&E staining of the collected femurs suggested that the area of trabecular bone was markedly reduced in OVX mice, whereas mice treated by CS-6 showed marked improvement of trabecular bone area (Figures 8A,C). TRAP staining showed obvious enhancement in the total number of TRAP^+^ OCs along the trabecular bone in OVX mice, whereas β-estradiol or CS-6 decreased the number of TRAP^+^ OCs per bone surface area (Figures 8B,D). However, the trabecular bone area and TRAP + OCs per bone surface area of OVX mice treated with a high dose of CS-6 did not differ from those of sham controls (both *p* > 0.05). The serum pro-inflammatory cytokines (IL-1β and TNF-α) and osteoclastogenesis serum markers, including RANKL, CTX-1, TRAcp5B, and OPG, were also measured in this study, and the results showed that OVX mice administrated with CS-6 showed a declined trend in the level of RANKL, CTX-1, TRAcp5B, IL-1β, and TNF-α and an increased tendency in the OPG level (Figures 8E–J). Notably, OVX mice with high-dose CS-6 also showed recovered level of serum pro-inflammatory cytokines (IL-1β and TNF-α) and osteoclastogenesis serum markers (RANKL and OPG) compared with the sham group (all *p* > 0.05). Thus, these data suggested that CS-6 could exert anti-inflammatory and anti-osteoporotic effects in OVX mice. It is notable that no adverse event occurred in mice treated with low- and high-dose CS-6 during the whole experiment, which indicated that CS-6 may possess a relatively safe drug profile.

Furthermore, we also investigate the effect of CS-6 on OB differentiation *in vitro* and *in vivo*, and found no positive results between OVX and CS-6 treatment groups. As shown in Supplementary Figure S2, CS-6 showed no obvious effects of the cell viability of bone marrow–derived stroma cells under indicated concentrations (BMSCs). (Supplementary Figure S2A). Additionally, CS-6 did not influence the alkaline phosphatase activity at 7 days and mineralization activity at 21 days during osteogenic differentiation (Supplementary Figure S2B). Next, results of *in vivo* experiment showed that no changes were observed among the OVX group, OVX with β-estradiol injection group, and OVX with CS-6 group in terms of cortical bone parameters, such as Ct. Ar/Tt.Ar and Ct. Th (Supplementary Figure S2C).

## Discussion

The nature of bone homeostasis is the coordination between the OBs and OCs. However, excessive activation of OCs may contribute to imbalances of bone homeostasis, constantly followed by various osteolytic diseases (Bertuglia et al., 2016; Raynaud-Messina et al., 2019; Wang et al., 2019). Thus, timely and appropriate medical therapy is necessary for those patients. In recent years, increasing medicines have been recommended to help ameliorate the effect imposed by overactivation of bone-resorbing activity, such as bisphosphonates (Green, 2003), pamapimod (Zhao et al., 2019), cathepsin K inhibitors (Mukherjee and Chattopadhyay, 2016), and alendronate therapies (Park et al., 2020). However, these therapies exhibit a series of side effects, including increased risk of osteonecrosis, renal toxicity, and allergic reaction, and may not be recommended for long-term administration (Hinchy et al., 2013; Lungu et al., 2018).

TCM has become increasingly popular due to its favorable effectiveness in treating a variety of orthopedic diseases (He et al., 2019; Wang et al., 2019). In fact, there have been numerous classical drugs for the treatment of bone loss and fracture diseases in clinical practice, such as *Gynochthodes officinalis* (F. C. How) Razafim. and *Curculigo orchioides* Gaertn (Nie et al., 2013; Wu et al., 2015). CS-6, as the major derivative of bufadienolides, has been generated from the postauricular glands and skin of *Bufo bufo* gargarizan Cantor, accounting for 1.75–5% of the extract, with significant antitumor effect (Banuls et al., 2013). Previous study has indicated the anti–bone-resorbing effect of CS-6 in multiple myelomas (Yu et al., 2014). However, knowledge is limited regarding the potential mechanism, and determining the underlying mechanisms of CS-6 underlying OCs formation is important for its treatment of bone loss. Our study is the first to explore the biological effects of CS-6 on osteoclastogenesis and OVX-induced bone loss. The salient findings from our present study revealed that CS-6 can mitigate osteoclastogenesis by suppressing the RANKL/RANK/TRAF6 cascades and ensuing translational activation of NFATc1 and c-Fos *via* inhibiting activation of NF-κB and ERK/MAPK signaling pathways, as well as down-regulating OC-related genes expression without significant cytotoxicity in the concentrations used. In addition, CS-6 can protect mice against OVX-induced bone loss, as indicated by the results of μCT, and histological analysis. The levels of RANKL, TRACP5b, CTX-1, and inflammatory cytokines, such as IL-1β and TNF-α, in mice serum were also effectively decreased by CS-6, whereas the OPG level increased. This present study provided the evidence that CS-6 has the potential to become a candidate for the therapy of osteolytic conditions mediated by elevated OC formation and bone resorption.

Mature OCs are defined by the varied morphological and functional characteristics, of which the positive TRAP staining and the formation of F-actin ring are the two key features (Xiong et al., 2018). In this present study, CS-6 significantly inhibited the number of TRAP^+^ multinucleated cells and the formation of F-actin ring without cytotoxicity over the entire period of osteoclastogenesis. Meanwhile, CS-6 treatment dramatically decreased the number and size of the F-actin ring in OCs following RANKL stimulation. What is more, CS-6 also had dramatic inhibitory effects on the RANKL-induced bone-resorbing activity. Conclusively, these findings demonstrated that CS-6 could effectively suppress the TRAP activity, the formation of F-actin ring, and bone-resorbing activity in RANKL-treated osteoclasts.

Over the period of the formation of functional OCs, bone-resorbing genes will be constantly activated, including TRAP, CTSK, DC-STAMP, and MMP9 (Lotinun et al., 2013; Chiu and Ritchlin, 2016; Collins et al., 2017; Xiong et al., 2018). Activated signaling cascades by RANKL are essential for successful differentiation of BMMs from OCs. After RANKL binding to its receptor RANK, BMMs would activate nuclear receptor NFATc1 and c-Fos, and the transcription of downstream OC marker genes. NFATc1 has been considered as a vital transcriptional regulator in OC differentiation (Takayanagi et al., 2002a; Sitara and Aliprantis, 2010). In addition, c-Fos is another essential regulator for osteoclastogenesis. It has been well established that c-Fos is also significantly important for the activation and expression of OC marker genes, including TRAP, CTSK, DC-STAMP, and MMP9 (Fleischmann et al., 2000; Takayanagi et al., 2002b; Wagner and Matsuo, 2003). Meanwhile, the expression of c-Fos could also affect the partial function of NFATc1 (Zhang et al., 2018). In the present study, CS-6 could attenuate the RANKL-induced expression of NFATc1 and c-Fos in mRNA and protein levels in both the early and late stages of osteoclastogenesis. What is more, the reduced expression of TRAP, CTSK, DC-STAMP, and MMP9 in mRNA or the protein level also confirmed the inhibitory effect of CS-6 on NFATc1 and c-Fos. Taken together, the results above suggest that CS-6 could suppress RANKL-induced functional OC formation by inhibiting the transcriptional activity of NFATc1 and c-Fos, and downstream OC-related genes expression. In addition, the decreased expression of OC-related genes by CS-6, combined with the reduced formation of F-actin and bone resorption pits, suggested the suppressive effect of CS-6 on OC differentiation *in vitro*.

The formation of functional OCs constantly undergoes cell proliferation, differentiation, cytoskeletal rearrangement, cell fusion, and final maturation. During the period of osteoclastogenesis, the NF-κB and MAPK (ERK, p38, and JNK) signaling pathways are first activated responding to the interaction between RANK and TRAF6 induced by RANKL (Boyle et al., 2003). When RANKL binds to its receptor RANK, TRAF6 will be recruited to form RANK/TRAF6 association (Liao et al., 2019). As shown in this study, CS-6 could decrease the RANKL-induced expression of TRAF6 in both early and late stimulation, which would lessen the interaction effect between RANK and TRAF6, and further affect downstream signaling pathways. Among these RANKL-initiated signaling pathways, the NF-κB pathway plays a crucial role during osteoclastogenesis (Vaira et al., 2008; Wu et al., 2015; Zhang et al., 2017). In general, NF-κB is kept in an inactive state in the cytoplasm due to the interaction with IκB family proteins which suppress the nuclear translocation of NF-κB (DiDonato et al., 1996). In fact, IκB kinase (IKK) complex is essential for the activation of NF-κB signaling. Once stimulated, the phosphorylated IKK-β activates IκB-α which becomes phosphorylated and ensue degraded, and thus, NF-κB will be released and translocate into cell nucleus (Li et al., 1998; Wu et al., 2019). CS-6 has been reported to affect the activation of the NF-κB pathway *via* inhibiting phosphorylation and activation of IKKβ (Yu et al., 2014). In the present study, we found that CS-6 could inhibit the phosphorylation of NF-κB and of IκB-α in cytoplasm, and immune-fluorescent staining also suggested the action of nucleus translation of NF-κB was also suppressed. Furthermore, computational docking analysis indicated that CS-6 could interact with IKKβ by bonding with its ATP-binding site, which may be the reason for the inhibitory effect of CS-6 on the IKKβ-IκBα-NF-κB cascade. However, the exact association of CS-6 and the IKKβ-IκB-α-NF-κB signaling pathway needs further investigation. Taken together, CS-6 could suppress the transcriptional activation of NF-κB pathways, at least in part, by blocking the activity of IKKβ and resultant IKKα degradation. In addition, the RANKL-induced phosphorylation of ERK signaling was also attenuated by CS-6 in this study. The decreased activation of NF-κB and ERK/MAPK signaling is in line with the decreased expression of NFATc1, c-Fos, and OC-related genes. In conclusion, the inhibitory effects of CS-6 on functional OC formation can in part be ascribed to the inactivation of NF-kB and ERK/MAPK signaling induced by RANK/TRAF6 interaction and consequent activation of c-Fos and NFATc1.

To confirm the effect of CS-6 on bone resorption *in vivo*, the OVX-induced bone loss mice model was established. Histological analysis validated the protective effects of CS-6 on OVX-mediated bone loss. As shown in Figure 7, OVX mice presented significantly reduced bone trabecula and increased numbers of TRAP^+^ OCs compared to the sham group, whereas this phenomena was reversed by β-estradiol or CS-6 administration. Notably, similar protective effects were seen between the OVX group with β-estradiol injected and the OVX group with high-dose CS6 injected (2.5 mg/kg). Thus, we believed that CS-6 may become a promising candidate for the OC-related disease, such as postmenopausal osteoporosis. Furthermore, microstructural analysis indicated by μCT demonstrated that CS-6 could prevent the decrease of the number and area of bone trabecula, and rescue the worsening bone quality of OVX mice. The serum level of RANKL, TRAP5b, and CTX-1 has been the typical indicator to evaluate the bone resorption activity *in vivo* (Kong et al., 1999; Shidara et al., 2008; Qi et al., 2019). On the other hand, OPG, as a member of the TNF receptor superfamily, could act as a soluble decoy receptor of RANKL *in vivo* and block the biological effects of RANKL (Han et al., 2018). Therefore, these biomarkers were measured in this study. As presented in the results, CS-6 administration significantly reduced the serum level of inflammatory factors (IL-1β and TNF-α) and osteoclastogenesis serum markers (RANKL, TRACP5b, and CTX-1), as well as increased the serum level of OPG, which further confirmed the protective effect of CS-6 on OVX-mediated bone loss.

Interestingly, *in vitro* experiments suggested that CS-6 show no obvious effect on OC differentiation and mineralization. In addition, no differences were observed regarding cortical bone parameters between OVX and CS-6 treatment groups, which may suggest that CS-6 show no direct effect on bone formation. However, the effect of CS-6 on OBs and bone formation still deserves further investigation. In addition, we did not perform an extensive study investigating the effects of CS6 on other bone, and bone marrow–derived cells would be conducive to determining the potential side effects of CS-6 treatment, which became one limitation of this study.

**FIGURE 9:**
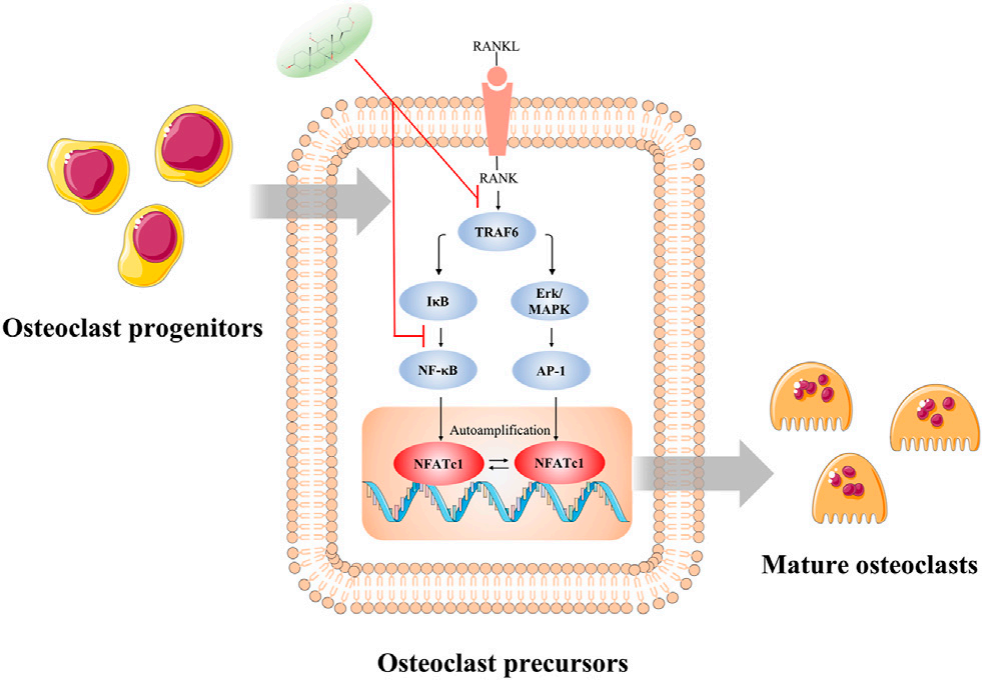
Illustration of the potential mechanism concluded by this study. The inhibitory effect of CS-6 on functional OC formation may be in part ascribed to the inhibition of NF-kB and ERK/MAPK signaling *via* lessening the binding of TRAF6 to RANK and consequently the activation of c-Fos and NFATc1. *p < 0.05, **p < 0.01, ***p < 0.001, ****p < 0.0001.

In conclusion, our present study has provided compelling evidence for the inhibitory effect of CS-6 on the formation of functional OCs. First, CS-6 could suppress RANKL-induced osteoclastogenesis from BMMs and ensue bone-resorbing activity *in vitro* with less cytotoxicity. Second, the anti-osteoclastogenic effect of CS-6 may be in part ascribed to the inactivation of NF-kB and ERK/MAPK signaling *via* lessening the binding effect of TRAF6 to RANK and consequently the activation of c-Fos and NFATc1 (Figure 9). Third, further OVX-induced bone loss mice model experiment confirmed that CS-6 with high dose could alleviate OC-mediated inflammatory response and estrogen-deficiency induced bone loss *in vivo*. Collectively, the data of this study above demonstrated that CS-6 with high dose could reverse trabecular bone volume in estrogen-deficient mice by decreasing osteoclast activity, and that CS-6 may be a potential candidate for further treatment of OVX-induced bone loss. However, there is still a lot of work that would need to be done before we could know whether CS-6 was efficacious as a disease treatment.

## Data Availability

The raw data supporting the conclusion of this article will be made available by the authors, without undue reservation, to any qualified researcher.
