# Supplementary material for: Gamabufotalin Inhibits Osteoclastgenesis and Counteracts Estrogen-Deficient Bone Loss in Mice by Suppressing RANKL-Induced NF-κB and ERK/MAPK Pathways
Source: Front Pharmacol. 2021 Apr 23;12:629968. doi: 10.3389/fphar.2021.629968 (PMC8104077; doi:10.3389/fphar.2021.629968)
Supplement: Supplementary file 1 [file Table1.docx]

| Targeted Gene | Forward (5’-3’) | Reverse (3’-5’) |
| --- | --- | --- |
| GAPDH | TGACCACAGTCCATGCCATC | GACGGACACATTGGGGGTAG |
| c-Fos | CCAGTCAAGAGCATCAGCAA | AAGTAGTGCAGCCCGGAGTA |
| NFATc1 | CCGTTGCTTCCAGAAAATAACA | TGTGGGATGTGAACTCGGAA |
| TRAP | CTGGAGTGCACGATGCCAGCGACA | TCCGTGCTCGGCGATGGACCAGA |
| CTSK | CTTCCAATACGTGCAGCAGA | TCTTCAGGGCTTTCTCGTTC |
| MMP-9 | AGTTTGGTGTCGCGGAGCAC | TACATGAGCGCTTCCGGCAC |
| DC-STAMP | TCCTCCATGAACAAACAGTTCCAA | AGACGTGGTTTAGGAATGCAGCTC |
| β3 -Integrin | TGACATCGAGCAGGTGAAAG | GAGTAGCAAGGCCAATGAGC |

**Supplementary Table S1 Oligonucleotide primers used for real-time PCR.**
