# Supplementary figures and images for: Gamabufotalin Inhibits Osteoclastgenesis and Counteracts Estrogen-Deficient Bone Loss in Mice by Suppressing RANKL-Induced NF-κB and ERK/MAPK Pathways
Source: Front Pharmacol. 2021 Apr 23;12:629968. doi: 10.3389/fphar.2021.629968 (PMC8104077; doi:10.3389/fphar.2021.629968)

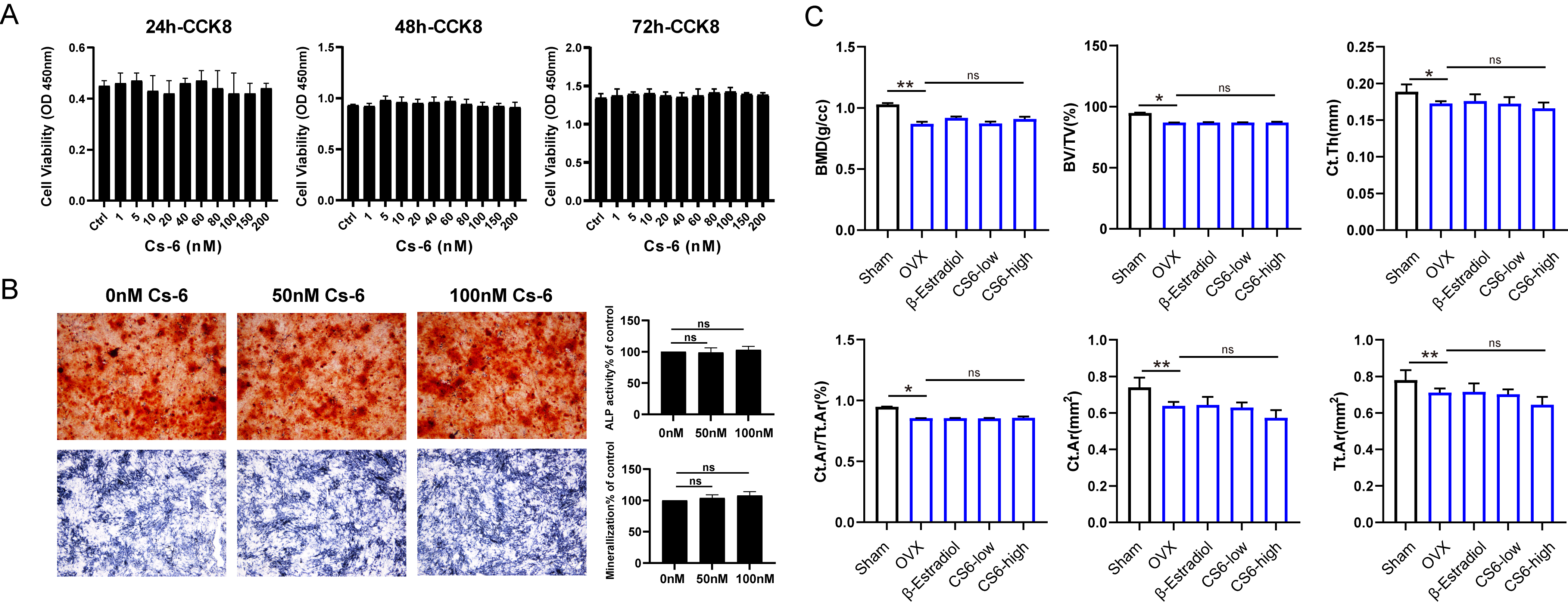

Supplement: Supplementary file 2 [file Image2.TIF]

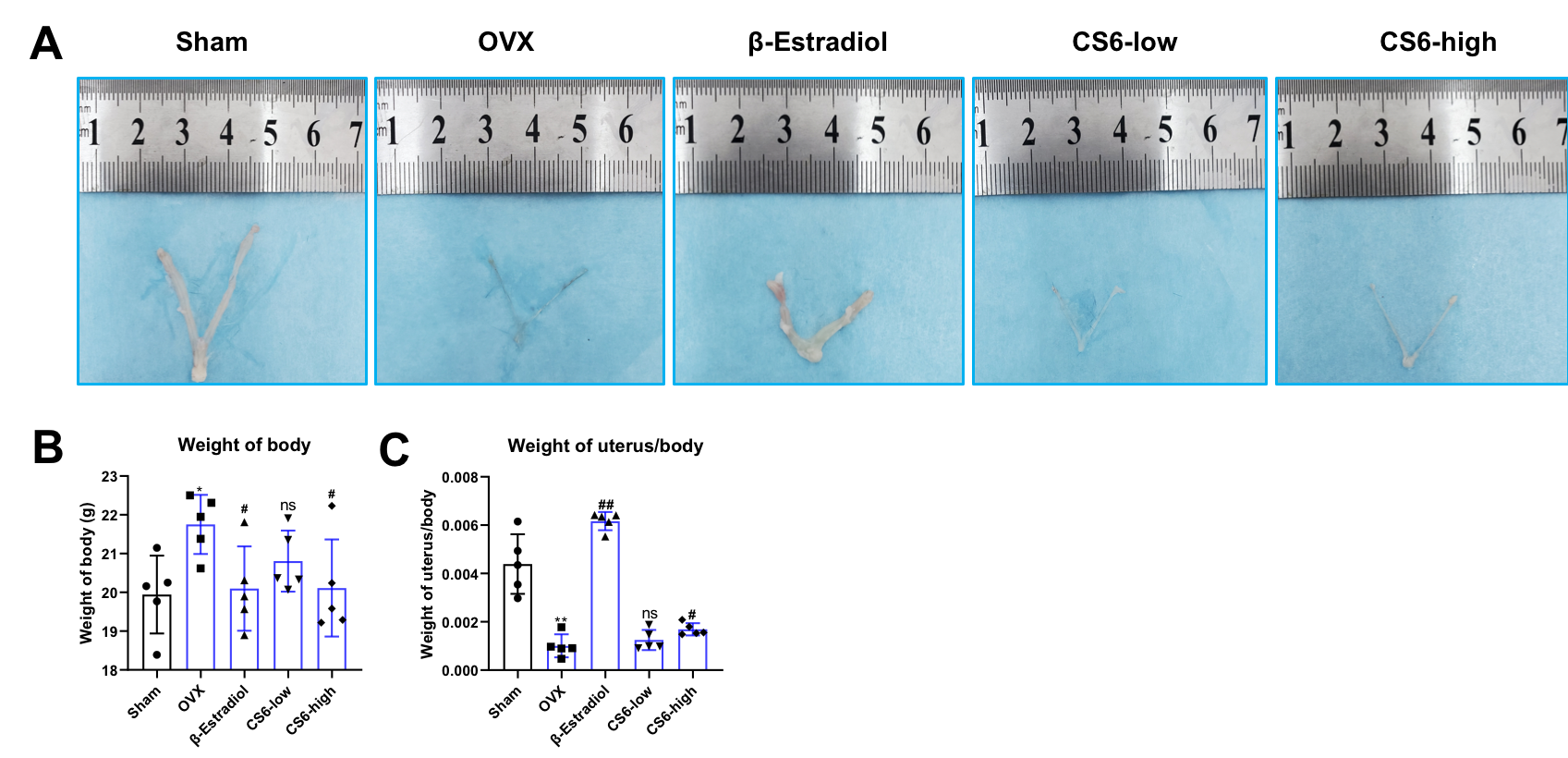

Supplement: Supplementary file 3 [file Image1.PNG]
